# The East Tennessee Record of Medicine and Surgery

**Published:** 1852-07

**Authors:** 


Art. VI.—*The East Tennessee Record of Medicine and Surgery.* Edited by Frank A. Ramsey, A. M., M. D. Knoxville: 1852.

Dr. Ramsey, the editor, has shown himself worthy of success in this first number of his journal. It bears the most unquestionable marks of industry and ardent professional zeal. He has our best wishes—we wish him all he expects or hopes from his enterprise, and would not add a word to damp his praiseworthy ardor. A large part of this number is from the pen of the editor. His report on the diseases of East Tennessee is an elaborate paper, and his editorials are spirited and manly.

